# Translumbar and Transhepatic Catheters for Hemodialysis in Chronic Kidney Disease: A Systematic Review and Meta-Analysis

**DOI:** 10.1016/j.xkme.2025.101216

**Published:** 2025-12-13

**Authors:** Rivaldo José Melo Tavares, Mariana Póvoa-Corrêa, Iandy de Souza Mateus Tarricone, Simone Collopy, Roberta Lins Gonçalves, Carlos Alberto da Silva Magliano, Gaudencio Espinosa Lopez

**Affiliations:** 1 Clementino Fraga Filho University Hospital, Federal University of Rio de Janeiro (UFRJ), Rio de Janeiro, RJ, Brazil; 2 Institute of Medical Science, Federal University of Rio de Janeiro, Macaé, RJ, Brazil; 3 D’Or Institute for Research and Education (IDOR), Rio de Janeiro, RJ, Brazil; 4 National Institute of Cardiology (INC), Rio de Janeiro, RJ, Brazil; 5 Pedro Ernesto University Hospital, Rio de Janeiro State University (UERJ), Rio de Janeiro, RJ, Brazil; 6 Physical Education and Physiotherapy College, Federal University of Amazonas (UFAM), Manaus, AM, Brazil

**Keywords:** Chronic kidney disease, translumbar access, transhepatic access, access exhaustion, hemodialysis, translumbar catheter, transhepatic catheter, kidney failure

## Abstract

**Rationale & Objective:**

Patients with chronic kidney disease (CKD) often reach a point where their options for hemodialysis access are exhausted, when transhepatic and translumbar access becomes an option. The aim of this study is to compare the prevalence of complications associated with both types of catheters through a systematic review and meta-analysis.

**Study Design:**

Literature-based systematic review and meta-analysis were accomplished in 2021/2022. Studies were obtained from 11 registries, including Medline/PubMed, Embase, and Scopus.

**Setting & Study Populations:**

Included studies involved patients with CKD in access exhaustion who underwent translumbar or transhepatic catheter placement.

**Selection Criteria for Studies:**

Eligible designs included clinical trials, quasi-experimental studies, observational studies, and case series; case reports were excluded.

**Data Extraction:**

Two independent researchers used a tailored sheet to extract data from the studies.

**Analytical Approach:**

A fixed-effect model for proportions was used to assess complications across 18 observational studies involving 649 catheters.

**Results:**

Compared to the translumbar group, the transhepatic group showed significantly higher proportions per 100 catheter-days of irreversible infections (0.085 [95% CI, 0.051-0.118] vs 0.015 [95% CI, 0.007-0.023]; *P* < 0.001) and irreversible dysfunction (0.259 [95% CI, 0.205-0.313] vs 0.071 [95% CI, 0.054-0.089]; *P* < 0.001). Total infections (*P* < 0.001), thrombosis (*P* < 0.001), and catheter displacement (*P* < 0.001) were also significantly more frequent in the transhepatic group.

**Limitations:**

The main challenge was the variability in study designs and the lack of randomized clinical trials, which was expected given the nature of the intervention.

**Conclusions:**

Translumbar access in CKD is associated with fewer complications; however, transhepatic access remains a viable option as a bridge to definitive access or transplantation.

Chronic kidney disease (CKD) is a growing burden to public health worldwide, being associated with high morbidity and mortality.^1^ In 2017, a prevalence of more than 850 million patients living with kidney disease was predicted, which is twice the prevalence of diabetes and 20 times the estimated number of HIV/AIDS patients.^2^ Considering this prevalence, 3.9 million patients were expected to be living with kidney replacement therapy in the same period.^2^ As hemodialysis (HD) is the most prevalent method in the United States,^3^ and usually the most common option to initiate dialysis therapy, secure vascular access is mandatory for long-term patient survival.

The vascular access required for HD presents a challenge in daily practice. Ideally, the best access should contemplate the patient’s needs while also being reliable and free of complications.^3^ Although arteriovenous fistulas and grafts are considered the first line access for hemodialysis, situations often arise where catheter-based access becomes necessary.^3^^,^^4^ However, traditional sites (internal and external jugular veins, subclavian veins, and femoral veins) might be exhausted by occlusions and complications, which can lead to the necessity of unconventional access, such as transhepatic (TH) and translumbar (TL) access, preferably with a long-term plan to a first-line access.^4^

Both unconventional catheters have a high proportion of mechanical complications compared to traditional ones.^4^ TL catheter involves accessing the infrarenal inferior vena cava through a percutaneous puncture along the lumbar vertebrae.^5^ On the contrary, TH access entails puncturing the middle hepatic vein to gain percutaneous access to the inferior vena cava and right atrium.^5^ As kidney replacement modalities are often combined,^6^ an individualized plan must be necessary, especially when kidney transplant is a possibility. Once inferior vena cava can be used as a possible site for a renal graft,^7^ the decision involving the catheter placement on upper or lower vena cava is an important resolution. Nevertheless, the choice between the TL or TH route to a dialysis access is not completely clear in literature, due to possible complications related to both catheters.

Therefore, the aim of this systematic review and meta-analysis is to evaluate the proportion of mechanical or infection complications (with or without catheter removal) in patients with chronic kidney disease and exhausted access sites for dialysis submitted to TH or TL access. The literature analysis will allow us to define proportions of dysfunction, infection, thrombosis, and catheter displacement in patients undergoing both procedures, enabling comparison of the occurrence of these complications with the 2 catheters analyzed.

## Methods

### Study Reporting and Registration

The protocol for this systematic review and meta-analysis was previously registered with the International Database of Prospectively Registered Systematic Reviews (PROSPERO; registration number: CRD42021265180). This study is reported according to the Preferred Reporting Items for Systematic Reviews and Meta-analyses guidelines.

### Population and Eligibility Criteria

The study population included adult patients with CKD and exhausted vascular access sites for dialysis. The intervention was a comparison between 2 kinds of catheters: TH catheter access and TL catheter access. Exhausted access sites for HD were defined as: necessity of vena cava access,^8^ impossibility of arteriovenous fistula formation,^9^ obstruction of the superior venous system (thoracic and brachiocephalic), and the use of a nonconventional access as a bridge to a definitive access.^4^^,^^10^

The exclusion criteria included: vascular access without HD as the primary purpose, studies lacking specific follow-up data (after at least one attempt to contact the corresponding author via official email), and studies reporting only minor complications (such as hematoma at the puncture site that does not interfere in the access function).

Studies were intended to be selected if they were clinical trials, quasi-experimental studies, or observational studies, and case series. Case studies were excluded. There were no date restrictions during the search process; however, only studies published in English, Portuguese, or Spanish were included.

### Outcomes

The primary outcome was the proportion of any complication leading to catheter disuse, prohibiting the patient from undergoing HD. The main occurrences in these cases were a significant decrease or obstruction of flow through the catheter, besides serious infections related to the access, treated with the catheter removal. Even when authors did not describe the cause of disuse, if catheter removal occurred, it was included as the primary outcome. In addition, complications that could be effectively treated with thrombolytics, catheter repositioning, or antibiotics were excluded from the primary outcome analysis.

The secondary outcomes include the raw data of average catheter follow-up time, the number of total catheter-days, the rate of dysfunction or infection (with catheter removal) per 100 catheter-days, and the rate of total infection, thrombosis, and displacement (with and without catheter removal) per 100 catheter-days. The endpoints were divided into 5 main groups: total infection (with and without catheter removal), total thrombosis, total displacement, infection with catheter removal, and dysfunction, which includes nonreversible thrombosis and displacement, and rare mechanical complications such as catheter kinks or accidental catheter removal.

### Literature Search and Study Selection

The systematic search was carried out in August 2021 from the following database: Medline (via Pubmed), LILACS (Latin American and Caribbean Health Sciences Literature), VHL Regional Portal (Virtual Health Library), Scielo (Scientific Electronic Library Online), Embase (from Elsevier), Scopus (from Elsevier), Web of Science (Clarivate Analytics), ASP (Academic Search Premier), CINAHL (Cumulative Index to Nursing and Allied Health Literature), Google Scholar and Cochrane Library. A complementary search was accomplished in July 2022 with the aim of updating literature findings. More details about the search strategy can be found on the registered protocol at PROSPERO.

After excluding duplicate studies with the Endnote program, the screened articles^11^ were evaluated by 2 independent reviewers using the Rayyan system.^12^ With this tool, both blinded reviewers evaluated titles and abstracts, considering the exposed inclusion and exclusion criteria. Every disagreement was resolved by consulting a third reviewer or through consensus between both previous reviewers.

### Data Extraction and Analysis of Bias

Data were extracted by 2 independent researchers using a standardized Excel sheet, which included study and patients’ baselines characteristics, as well as outcomes data. Bias analysis was conducted using the Robins I with specific template, defining the level of risk bias for each analyzed domain: confounding, selection of participants, classification of interventions, missing data, measurement of outcomes and selection of the reported result.

### Statistical Analysis

For the meta-analysis, studies were analyzed and grouped according to the type of the catheter studied (TL or TH access). The results of each study were summarized by group, including the number of analyzed catheters, median time of follow-up in days, and rate of complications per 100 catheter-days.

Pooled effects sizes calculations were performed using a fixed-effects model, without weighted proportion, using a Shiny Web Application for R package version 1.8.0.^13^ Confidence intervals were estimated using the binomial exact method. The analysis of pooled effects size considered 2 groups of catheter disuse as outcomes: infection leading to catheter removal and dysfunction resulting from mechanical occurrences leading to catheter removal. These results are represented as the proportion of events per catheter-days using forest plot graphics. Pooled effects sizes were compared using the Wald test for proportions, and results were expressed as *P* values. The same analysis was conducted for secondary outcomes (total infection, total thrombosis, and total displacement). Heterogeneity was calculated using Cochran's Q test, using the same R package version, with the result expressed as *P* value. Additionally, the I^2^ statistics were also predicted to assess the percentage of variation across studies.

## Results

### Search Results

**Figure 1:**
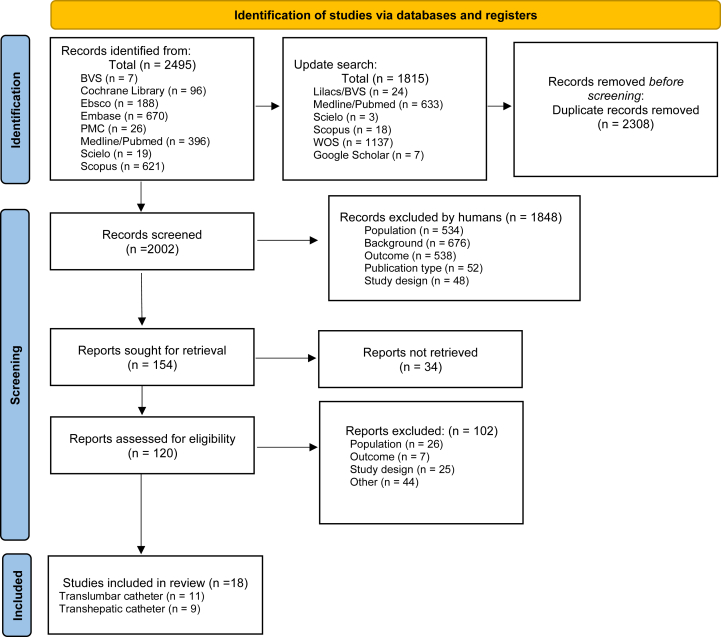
The flowchart shows the systematic design of literature search and the results of that search.

The search across all aforementioned databases identified 2,488 records in 2021, with an additional 1,822 articles identified in the 2022 update. After evaluating for duplicate articles, 2,308 records were excluded. From the remaining 2,002 studies, 1,848 articles were further excluded after evaluation of the title and abstract. Thus, 154 articles were sought for retrieval, and 120 studies were available for eligibility assessment. Finally, 18 studies were eligible to be included, of which 9 focused exclusively on TL access, 7 on TH access alone, and 2 addressed both TL and TH access. All included articles were observational studies or case series (Fig 1).

### Studies Characteristics

Table 1^8^^,^^10^^,^^14^, ^15^, ^16^, ^17^, ^18^, ^19^, ^20^, ^21^, ^22^ outlines the key data of the included studies related to TL catheters, including publication year, study type, and the number of catheters analyzed. The entire period covered by the selected studies was between 1995 and 2021. A total of 11 articles presented in this table were observational studies, outlined as cases series, transversal, or cohort studies. The number of catheters varied between articles, but with a total number of 359 catheters analyzed.

**Table 1:** TL Studies Data

Study	Number of Catheter	Median Time of Follow-Up (d)	Total Catheters-Day	Rate of Complications per 100 Catheters-Day				
				Total Infection	Infections With Catheter Removal	Dysfunction	Total Thrombosis	Total Catheter Displacement
Lund et al^14^ (1995)	17	125,000	2,121	0.280	0.140	0.120	0.330	No information
Biswal et al^15^ (2000)	13	173,230	2,252	0.000	No information	0.130	0.040	0.130
Assounga et al^16^ (2008)	9	333,300	3,000	0.130	0.000	0.100	0.033	No information
Power et al^17^ (2009)	39	406,760	15,864	0.280	0.010	0.050	0.060	0.020
Herscu et al^8^ (2012)	3	276,000	828	0.120	0.000	No information	0.000	No information
Nadolski et al^18^ (2013)	92	85,000	7,825	0.510	0.510	0.330	0.140	0.190
Kade et al^19^ (2014)	16	261,000	4,196	0.022	0.022	0.170	0.010	0.070
Liu et al^20^ (2015)	84	128,000	10,769	0.260	0.260	0.370	0.220	0.110
Moura et al^21^ (2018)	12	316,000	3,786	0.020	0.020	0.000	0.000	0.00
Mori et al^22^ (2019)	26	591,000	15,366	0.010	0.010	0.080	0.060	0.010
Jonszta et al^10^ (2020)	48	555,500	26,666	0.015	0.015	0.037	0.007	0.030

Similarly, Table 2^8^^,^^22^, ^23^, ^24^, ^25^, ^26^, ^27^, ^28^, ^29^ displays data from studies focusing on TH catheters. Of the 9 articles included, observational studies predominated, encompassing both cohort and cross-sectional designs. The period covered by the studies in this table ranged from 2003 to 2019, with a total of 290 catheters analyzed. Both tables also include extracted data from these studies, detailing the median follow-up time in days per catheter, the total catheter-days, the rate of primary complications per 100 catheter-days (including infections and dysfunctions resulting in catheter removal), and the rate of secondary complications per 100 catheter-days (comprising total infections, thromboses, and catheter displacements). A total of 92,773 catheter-days were recorded for TL catheters, whereas 33,724 catheter-days were recorded for TH catheters. Among the included articles, 2 provided data on both catheter types, 9 studies analyzed only TL devices, and 7 investigations were solely focused on TH catheters.

**Table 2:** TH Studies Data

Study	Number of Catheters	Median Time of Follow-Up (in Days)	Total Catheter-Days	Rate of Complications per 100 Catheter-Days				
				Total Infection	Infections With Catheter Removal	Dysfunction	Total Thrombosis	Total Catheter Displacement
Sanal et al^23^ (2016)	34	125,380	4,263	0.180	0.180	0.590	0.370	0.180
Motta et al^24^ (2010)	9	200,000	1,803	0.050	0.050	0.110	0.050	0.150
Younes et al^25^ (2011)	127	87,000	11,049	0.220	0.220	0.610	0.180	0.390
Herscu et al^8^ (2012)	6	305,600	1,834	No information	No information	0.109	0.050	0.050
Mori et al^22^ (2019)	14	236,000	3,297	0.060	0.060	0.120	0.090	0.030
Stavropoulos et al^26^ (2003)	36	24,500	873	0.220	0.220	2.520	2.40	0.550
Smith et al^27^ (2004)	51	138,000	7,038	0.040	0.040	0.260	0.120	0.070
Barros et al^28^ (2014)	4	221,250	885	0.110	0.110	0.220	0.110	0.110
Kwan et al^29^ (2019)	9	298,000	2,682	0.070	0.000	0.190	0.110	0.070

### Catheter-Related Infection

**Figure 2:**
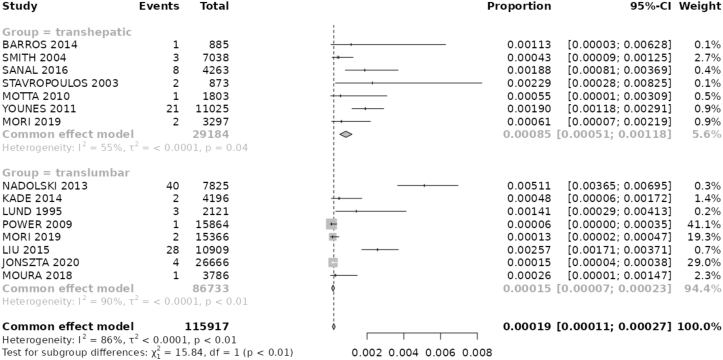
Forest Plot shows proportion of Infections with catheter removal (per catheter-day). Fixed-effect model demonstrates less irreversible infections on TL access. Total– number of catheter-days evaluated in the study.

Within the TH group, 9 articles provided infection data related to catheter usage. Across these studies, a total of 44 events were reported, with 38 events leading to catheter removal. Conversely, in the TL data, all articles provided information on total infections while 10 articles specifically addressed irreversible infections. Among these, a total of 132 total infection events were recorded, with 81 events leading to removal procedures. The pooled proportion of total infection at TH group was 0.086 (95% CI, 0.054-0.118) events per 100 catheter-days, compared with 0.026 (95% CI, 0.016-0.037; *P* value < 0.001) events per 100 catheter-days in the TL group. Regarding infection events resulting in catheter removal, TH studies presented a pooled proportion of 0.085 (95% CI, 0.051-0.118) events per 100 catheter-days, while TL studies showed a proportion of 0.015 (95% CI, 0.07-0.023; *P* value < 0.001) events per 100 catheter-days (Fig 2 and Table 3).

**Table 3:** Pooled Proportions of Catheter Complications

Complication	Transhepatic Proportion (per 100 Catheter-Days) (95% CI)	Translumbar Proportion (per 100 Catheter-Days) (95% CI)	*P*^a^
Total infection	0.086 (0.054-0.118)	0.026 (0.016-0.037)	<0.001
Infection with catheter removal	0.085 (0.051-0.118)	0.015 (0.007-0.023)	<0.001
Dysfunction	0.259 (0.205-0.313)	0.071 (0.054-0.089)	<0.001
Total thrombosis	0.129 (0.091-0.167)	0.020 9 (0.011-0.030)	<0.001
Total catheter displacement	0.097 (0.064-0.130)	0.029 (0.017-0.040)	<0.001

a Pooled effect sizes compared with Wild Test for proportions.

### Mechanical Complications

**Figure 3:**
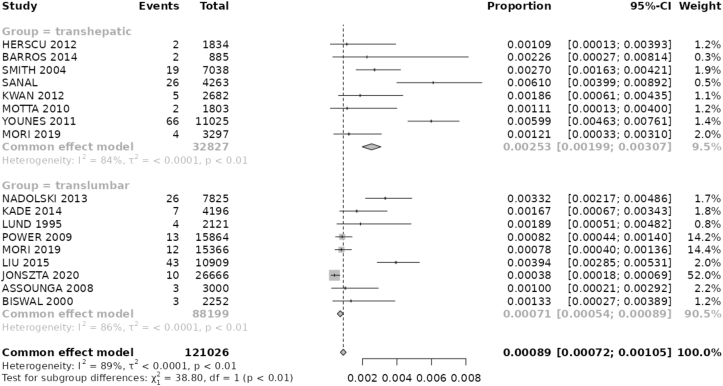
Forest Plot shows proportion of catheter dysfunction (per catheter-day). Fixed-effect model demonstrates less irreversible dysfunction on TL access. Total – number of catheter-days evaluated in the study.

Among the reviewed articles, only one did not provide data on removal due to dysfunction, all provided information about thrombosis, whereas 3 articles lacked information about catheter displacement without definitive catheter removal. In the TH studies, there were a total of 148 dysfunction events, 75 thrombosis without the need of catheter removal, and a total of 69 displacement events. In addition, in the TL articles, a total of 121 occurrences of dysfunction were reported, along with 69 thromboses and 48 catheter displacements. The calculated pooled proportion of dysfunction leading to catheter removal was 0.259 (95% CI, 0.205-0.313) events per 100 catheter-days in TH patients compared with 0.071 (95% CI, 0.054-0.089; *P* value < 0.001) events per 100 catheter-days in TL access (Fig 3 and Table 3). The proportion of total thrombosis was 0.129 (95% CI, 0.091-0.167) per 100 catheter-days in the TH group compared with 0.020 (95% CI, 0.011-0.030; *P* value < 0.001) events per 100 catheter-days in the TL studies. Similarly, the proportion of total catheter displacement was 0.097 (95% CI, 0.064-0.130) per 100 catheter-days in TH articles, compared to 0.029 (95% CI, 0.017-0.040; *P* value < 0.001) events per 100 catheter-days in the TL studies. These findings define the TL catheter as less prone to mechanical complications. Dysfunction exhibited a higher proportion of occurrence compared with infection leading to catheter removal within each group. In the TH group, dysfunction had a higher proportion than infection leading to removal (*P* value < 0.001), whereas in the TL group, dysfunction proportions were also higher than irreversible infection (*P* value < 0.001).

### Bias Analysis

**Figure 4:**
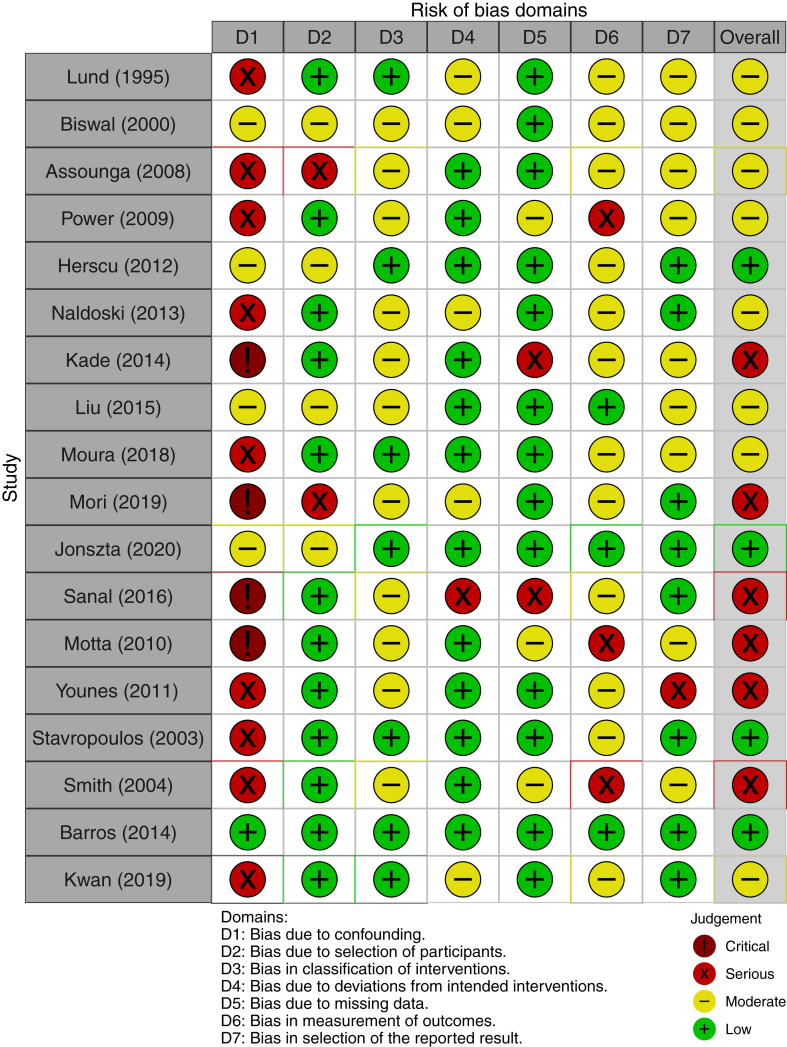
Risk bias analysis detailed by study and specific domains. Overall bias analysis in the last column.

**Figure 5:**
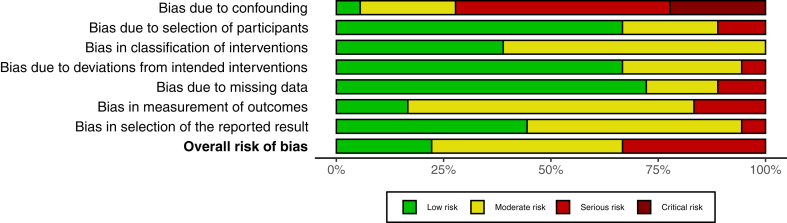
Summary of authors’ judgments about each risk-of-bias domain and overall risk of bias for all 18 included studies.

Detailed information about risk of bias for each study can be seen on Figure 4, whereas the summary assessment is presented on Figure 5, with a colored graph representing each domain. No study was judged to have an overall critical risk of bias. Even so, a serious overall risk of bias was encountered in 6 studies, especially related to a high risk of bias due to confounding. For the remaining 12 studies, the overall risk of bias was low/moderate.

## Discussion

This systematic review and meta-analysis evaluated 18 articles, with 7 studies presenting all evaluated outcomes related to TL catheters and 9 related to TH catheters. Irreversible dysfunction was evaluated by 9 TH studies and 8 TL articles. A significant difference between the proportion of dysfunction for both catheters analyzed was observed, favoring TL catheters with fewer occurrences than TH access. Similarly, 7 studies presented infection data (with catheter removal) on TH group and 8 on TL articles, with a significant difference in the proportion of this infection, also in favor of TL catheter.

Even when considering secondary complications (total infection, total thrombosis, and total catheter displacement), a difference in proportions was observed, favoring TL catheters. These results align with literature observations regarding TH access, which often exhibit a higher rate of complications, especially due to thrombosis and displacement.^26^ Some authors suggest that the short vessel path in this case may contribute to these complications,^26^ which do not occur with the direct puncture of the retrohepatic vena cava. It has even been suggested that TH access should only be used after a TL access failure or as a bridge to a permanent access,^9^^,^^19^^,^^26^ given the recurring complications associated with this catheter type. Another potential rationale for preferring the TH route, as suggested by some authors, used to be the concern regarding hemorrhagic complications resulting from catheter removal. This is because bleeding in such situations can often be effectively controlled through venous tract embolization.^25^^,^^30^ In addition, the TH catheter offers the advantage of an easier revision technique, which is particularly valuable given that fibrosis in the retroperitoneal region can complicate TL revisions when necessary.^25^ However, it appears that the primary factor influencing the decision to use the TH route is often the preference of the interventional radiologist or the patient's anatomical characteristics.^26^

Although some authors advocate for the security and functionality of TH access,^27^ the most significant complication associated with TH access varies across studies.^23^^,^^25^, ^26^, ^27^ Nonetheless, thrombosis and catheter displacement consistently emerge as primary concerns, as mentioned previously. The short distance between the right atrium, the inferior vena cava, and hepatic vein, may contribute to catheter migration.^25^ In addition, thoracic respiratory movements, in addition to coughing and abdominal distension, also negatively impact the stability of TH catheters, favoring migration and displacement.^25^ Our findings, which indicate a higher proportion of catheter dysfunction compared with infection leading to catheter removal, are consistent. However, they are at odds with the study by Liu et al,^20^ which reported infection as the most frequent complication.

The catheter management aiming at its maintenance is highly cited in specific literature of this subject, which aligns with the findings of our evaluated studies. For instance, Lund at al^14^ used chemical thrombolysis in 7 patients, achieving success in 5 cases. Similarly, Power et al^17^ presented a higher number of infections than the total number of catheters analyzed, suggesting that antibiotic therapy was effective in resolving some infection cases, thus enabling the maintenance of the catheter. These authors assigned the high level of infections to the site of TL access evaluated in their study, contrasting with the relatively lower impact of infection observed in our meta-analysis compared to irreversible dysfunction. Additionally, Moura et al^21^ registered no infection requesting catheter removal, and also mechanical complications with low flow were resolved either through contrast studies or thrombolysis.

According to KDOQI 2021,^3^ there must be a contingency plan for HD access, which means an ideal conduct for saving and remediation of an access problem. Conversely, a succession plan exists to guide the strategy for the next access in the event of failure or the need to interrupt the current catheter.^3^ Both strategies are frequently mentioned in the evaluated studies when an exhausted access scenario occurs, always looking at a contingency plan with the perspective of a succession plan, which is the guidance through a definitive access. In this perspective, TL catheter implantation is considered as a safe and effective technique for enabling HD access when upper body veins are occluded.^10^ This enables continuity of proper treatment and facilitates the exploration for a potential new definitive access.^10^ When compared with the complication rates of default access via upper body veins, the long-term patency and complication rates are similar.^28^ Despite assertions by some authors regarding the viability of TH catheters as an option for exhausted dialysis access,^25^^,^^27^ our data, allied with previous literature, suggest otherwise. TH access tends to lack long-term viability.^28^ However, it is important to bear in mind that authors inclined to consider TH catheter as a contingency strategy, in fact, defended a methodological approach that considers previous exhaustion of all access sites or the objective to preserve a venous axis as TH access indication.^25^^,^^27^ This approach was not consistently followed by TH studies, which often mixed indications for patient selection, thereby impacting the final results.^26^

The eligibility criteria for selecting an unconventional access can vary significantly. Perhaps, the lack of a formal definition and specific criteria of exhausted access in dialysis seems to be a barrier to providing guidance on selecting which unconventional catheter should be preferred for each case. This is likely due to anatomical and clinical peculiarities. For example, a patient with a venous territory suitable for creating an arteriovenous fistula may have a compromised arterial site, limiting the confection of the fistula. Even economic or social limitations can influence access decisions,^31^ alongside the experience of the medical team, which may limit the range of therapeutic options available to patients with exhausted access.

Although our data demonstrates the superiority of TL catheters as unconventional access, particularly in terms of device longevity due to lower proportions of complications, we acknowledge that the TH route may still have a role in cases of exhausted access. In this perspective, TH access could be considered as a short-term strategy, particularly in medical services with an active and effective kidney transplant program, as the vena cava can be considered a potential site for kidney graft placement.^7^ Therefore, especially on the context of living donor kidney transplantation, which is an elective procedure, TH access seems to be highly appealing. This preference in these cases stems from the smaller territory occupied by the vena cava when compared with TL access,^4^ allowing for a more comfortable vein territory for renal graft implantation. Nevertheless, it is important to take into account structural issues and the support capacity of each health care service, as frequent complications lead to the need for multiple revisions and catheter exchanges.^23^^,^^26^

As already exposed, the lack of a formal definition of exhausted access for dialysis posed a significant limitation to meta-analyzing the available data. However, we dealt with this issue by defining exhausted access with a broad practical concept, to contemplate the most used concept presented in the literature.^7^ Yet, high heterogeneity was also a challenge, as expected. We had to deal with different methods of data collection, varying sample sizes, diverse record formats, and varying follow-up periods. This qualitative perception of heterogeneity is supported by our data, with Cochran’s Q test yielding *P* values < 0.05 and I^2^ Statistic indicating a high percentage of variance across studies (Figs 2 and 3).

It is important to highlight that some studies did not track their catheters until the conclusion of the study. Thus, patients who received definitive access (arteriovenous fistula or transplant) or who died while using the catheter were excluded from the analysis, following the methodology previously published by Liu et al.^20^ Another issue was the lack of information in some studies regarding the final patient outcomes (such as death or definitive access) as well as the presence of potential confounders,^14^^,^^28^ such as specific medical conditions or the use of drugs that can influence catheter function. For example, some studies documented the presence of conditions like antiphospholipid syndrome, Factor V Leiden deficiency, and even hyperhomocysteinemia,^8^^,^^25^ while some patients used coumarins or aspirin alone or even a combination of both.^20^^,^^25^

To our knowledge, this is the first meta-analysis to compare the use of TH and TL catheters for dialysis in the context of patients with exhausted access. TL catheters exhibited a lower proportion of device removal than TH catheters due to dysfunction and infection. Even when considering total complications (with and without catheter removal), TL access demonstrated superiority. However, TH catheters seem to have a role when a kidney transplant is the short-term goal. In this regard, and considering the favorable long-term outcomes of kidney transplants, well-designed studies that consider this goal as a possible outcome are needed in the future.

## Conclusion

Based on the systematic review and meta-analysis conducted, it can be concluded that patients with CKD and exhausted access sites for dialysis can benefit from both TL and TH catheter access. The TL catheter access demonstrated fewer complications, both reversible and irreversible, compared with TH catheter access. However, TH catheter access can serve as a bridge to a definitive access for kidney transplant, making it a valuable option in certain clinical scenarios. Further studies focusing on specific goals, such as kidney transplant outcomes and long-term viability of TH catheter access, are needed to provide more comprehensive insights into the optimal management of patients with CKD with exhausted access sites.
